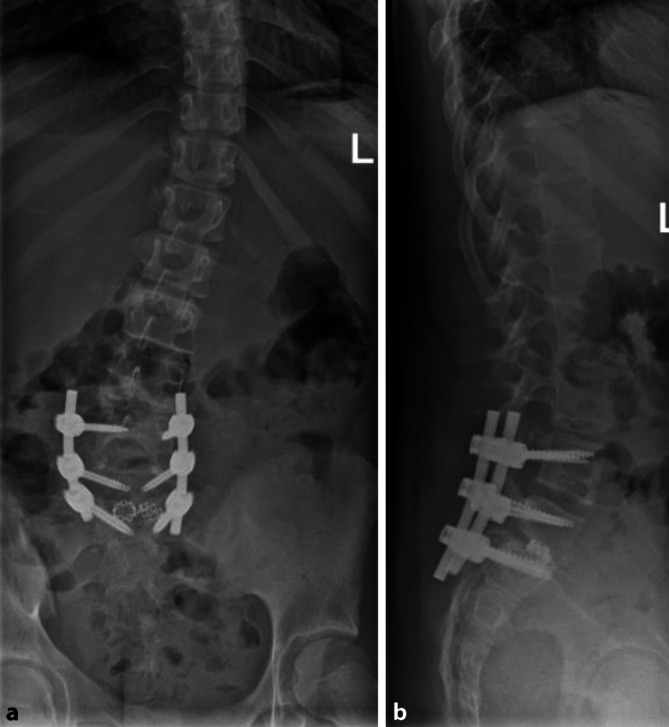# 13/w – Leichte Schiefhaltung und ins Bein ausstrahlender Rückenschmerz: Vorbereitung auf die Facharztprüfung: Fall 37

**DOI:** 10.1007/s00132-020-04019-y

**Published:** 2020-10-12

**Authors:** Lorenz Wanke-Jellinek, Christoph Mehren

**Affiliations:** 1 Schön Klinik München Harlaching, Harlachinger Straße 51, 81547 München, Deutschland; 2 Paracelsus Medical University, Salzburg, Österreich

**Keywords:** Spondylolisthese, Anterolisthese, Spondyloptose, Spondylolisthesis vera, Radikulopthie

## Prüfungssimulation

### Fallschilderung

Die 13-jährige Patientin stellt sich in Begleitung ihrer Eltern in der Sprechstunde vor. Sie berichtet über gering ausgeprägte Rückenschmerzen sowie linksseitig ausstrahlende Beinschmerzen, die seit einigen Monaten bestehen und im Verlauf eher zunehmen würden. Ein Trauma sei nicht erinnerlich. Den Eltern ist zudem eine eher zunehmende Schiefhaltung der Tochter aufgefallen.

## Prüfungsfragen

- Welche weiteren Fragen stellen Sie anamnestisch? Was sind relevante klinische Untersuchungen?
- An welche Erkrankungen denken Sie differenzialdiagnostisch? Welche Untersuchungen würden Sie zur weiteren Abklärung durchführen?
- Beschreiben Sie die Ätiologie des Krankheitsbildes und bekannte Risikofaktoren, die zum Auftreten des Krankheitsbildes führen können.
- Welche Klassifikationen zur Einteilung des Krankheitsbildes können Sie nennen?
- Welche Therapieoptionen bestehen generell zur Behandlung des Krankheitsbildes?
- Welches Vorgehen empfehlen Sie im vorliegenden Fall und warum?

### Antworten

#### Welche weiteren Fragen stellen Sie anamnestisch? Was sind relevante klinische Untersuchungen?

##### Anamnese.

Seit wann bestehen die Beschwerden? Traten sie akut oder schleichend auf? Ist die Patientin sportlich aktiv? Besteht der Hinweis auf das Vorliegen eines Infekts (Fieber, subjektives Befinden)? In welcher Situation treten die Beschwerden besonders auf (Belastung, monotone Haltung)? Besteht ein subjektives sensibles oder motorisches Defizit der Extremitäten? Bestehen Blasen- oder Mastdarmstörungen? Bestehen bekannte Vorerkrankungen (Spina bifida)?

##### Der Fall.

Die Beschwerden wären vor einigen Monaten schleichend ohne vorangehendes Trauma aufgetreten. Das Mädchen betreibt nur Schulsport. Es bestünde kaum Rückenschmerz, der Beinschmerz links würde sich nach Sport oder längerem Stehen verschlechtern. Anamnestisch kein Hinweis auf Infekt oder B‑Symptomatik, kein subjektives sensomotorisches Defizit. Keine relevanten Vorerkrankungen bekannt.

##### Klinische Untersuchung der Wirbelsäule.

- Inspektion im Stehen und bei maximal möglicher Inklination. Findet sich eine Hyperlordose der Lendenwirbelsäule (LWS)? Besteht der Hinweis auf eine Skoliose?
- Inklination/Reklination/Seitneigung eingeschränkt?
- Besteht Klopf- oder Druckschmerz? Stufenbildung der Proc. spinosi?
- Im Liegen: besteht eine Hüft-Lenden-Strecksteife?
- Besteht ein radikulär zuordenbarer Schmerz der unteren Extremität? Lasègue-Test positiv?
- Grobe Prüfung der Kraft und Sensibilität der unteren Extremität.

##### Der Fall.

Es findet sich ein unauffälliger Lokalbefund der Haut, die Wirbelsäule steht im Lot. Es besteht ein geringer Schultertiefstand rechts. Die Taillendreiecke sind gering asymmetrisch, in Vorneigung findet sich ein geringer Rippenbuckel und Lendenwulst links. Es besteht kein Druck- oder Klopfschmerz der Wirbelsäule oder der Iliosakralgelenke. Die In‑/Reklination sowie Seitneigung sind frei durchführbar. Der Finger-Boden-Abstand beträgt ca. 20 cm. Der linksseitige Beinschmerz entspricht am ehesten dem Dermatom L5 (lateraler Ober- und Unterschenkel). Es besteht eine Hüft-Lenden-Strecksteife. Es besteht eine Hypästhesie im Bereich des rechten Fußrückens, jedoch kein motorisches Defizit der unteren Extremitäten. Die Kraft ist seitengleich 5/5. Zehenspitzen- und Fersengang sind ebenso wie eine Kniebeuge frei vorführbar.

#### An welche Erkrankungen denken Sie differenzialdiagnostisch? Welche Untersuchungen würden Sie zur weiteren Abklärung durchführen?

##### Differenzialdiagnosen.

- Bandscheibenpathologie mit Nervenwurzelkompression
- infektiöse Erkrankungen der Wirbelsäule (Spondylitis/-diszitis)
- Tumorerkrankungen/Metastasen

##### Weiterführende Untersuchungen.

- Röntgen der LWS a.-p., seitlich sowie ggf. Schrägaufnahme (Beurteilung der Pars interarticularis)
  *Zwischenfrage:* Wie kann man eine Spondylolisthesis vera von einer degenerativen Olisthese L5/S1 einfach im Röntgen unterscheiden? *Antwort:* seitliches Röntgen der LWS: Stufe am Dornfortsatz L4/5 → Olisthesis vera L5/S1 mit Lyse Lendenwirbelkörper (LWK) 5, Stufe am Dornfortsatz L5/S1 → degenerative Olisthese L5/S1
- MRT LWS: Goldstandard zur Beurteilung der neuralen Strukturen und des knöchernen Defekts. Beurteilung einer frischer Stressfraktur möglich.
- CT LWS: Alternativ, wenn eine MRT nicht möglich ist; ggf. ergänzend präoperativ; Abwägung, ob eine Frakturheilung bereits stattfindet, oder ob ggf. diese nicht mehr eintreten wird (Sklerosierung der Frakturränder → Ruhigstellung nur bis zur Schmerzreduktion, da man nicht mehr von einer konservativen Frakturheilung ausgehen kann).
- Nervenleitgeschwindigkeit: zur Abgrenzung eines chronischen gegenüber akuten Nervenschadens.
- Myelographie und Knochenszintigraphie sind nur in Ausnahmefällen indiziert.

##### Der Fall.

**Figure 1:**
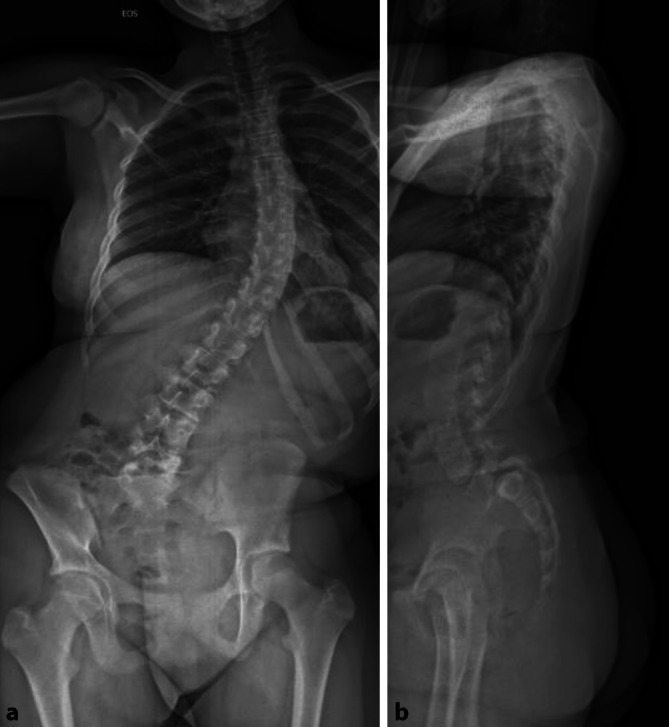

Bei der Patientin wurde ein Röntgen in 2 Ebenen durchgeführt. Dabei fand sich eine hochgradige Anterolisthese des LWK 5 mit konsekutiver linkskonvexer Skoliose (Abb. 1a, b). Im weiteren Verlauf wurden zusätzlich eine MRT und CT der LWS veranlasst. Aufgrund von fehlenden klinischen Infektzeichen und B‑Symptomatik wurde zunächst auf eine Laboruntersuchung verzichtet.

#### Beschreiben Sie die Ätiologie des Krankheitsbildes und bekannte Risikofaktoren, die zum Auftreten des Krankheitsbildes führen können.

- Es besteht eine genetische Prädisposition, v. a. für die dysplastische Spondylolisthese.
- Vermehrte körperlicher Belastung in Hyperlordose und rezidivierende Mikrotraumen in der Kindheit/Jugend [1] beim Sport erhöhen das Risiko des Auftretens (Risikosportarten: Turnen, Speerwerfen, rhythmische Sportgymnastik, Ringen, Gewichtheben).
- traumatisch
- degenerativ

#### Welche Klassifikationen zur Einteilung des Krankheitsbildes können Sie nennen?

##### Radiologische Einteilung nach Meyerding.

- Grad 1: Abrutschen um bis 25 % des Wirbelkörpers
- Grad 2: Abrutschen um bis 50 % des Wirbelkörpers
- Grad 3: Abrutschen um bis 75 % des Wirbelkörpers
- Grad 4: Abrutschen um bis 100 % des Wirbelkörpers
- Grad 5: Vollständiges ventrales Abkippen des Wirbelkörpers („Spondyloptose“)

##### Wiltse-Klassifikation [2] der Spondylolisthese nach Ätiologie.

- I. dysplastisch: Kongenitaler Defekt der hinteren Säule mit Insuffizienz der Facettengelenke. Die Pars interarticularis ist intakt
- II. isthmisch: Es besteht ein Defekt der Pars interarticularis; häufigste Form.
- III. traumatisch
- IV. pathologisch
- V. postoperativ/Iatrogen

##### Marchetti-Bartolozzi-Klassifikation der Spondylolisthese [3].

- kongenital:
  - ausgeprägt dysplastisch
  - gering dysplastisch
- erworben:
  - traumatisch
  - postoperativ
  - pathologisch
  - degenerativ

##### Mac-Thiong-Labelle-Klassifikation [4].

- Kombiniert Meyerding-Klassifikation mit Ausprägung der Dysplasie und spinopelvinen Parametern.
- Klassifikation ergibt Therapieempfehlung.

##### Der Fall.

Bei dem Mädchen fand sich in der Anamnese kein Hinweis auf vermehrte sportliche Betätigung bzw. auf Risikosportarten. Die radiologische Bildgebung ergab das Vorliegen einer isthmischen Spondylolisthese von LWK 5, Grad 4 nach Meyerding. In der durchgeführten MRT fand sich eine geringe Reposition der Spondylolisthese LWK 5 auf Grad 3 nach Meyerding.

#### Welche Therapieoptionen bestehen generell zur Behandlung des Krankheitsbildes?

- Die Therapie ist abhängig von Ausprägung der Spondylolisthese, Beschwerden und Patientenalter.
- Generell: Bei Spondylolisthese Meyerding-Grad 1 und 2 sollte primär eine konservative Therapie erfolgen.
- Unilaterale Spondylolyse: gute Prognose unter konservativer Therapie.
- Bei Kindern/Jugendlichen mit akuter Symptomatik:
  - Unterscheidung uni-/bilaterale Lyse mit/ohne Anterolisthese.
  - Bei unilateraler Spondylolyse: Häufig kontralaterale Beteiligung im Verlauf. Wenn in der CT keine Sklerosierung der Frakturspalte sichtbar ist, bestehen gute Heilungschancen unter konservativer Therapie.
  - Initiale Sportkarenz bei akuter Symptomatik, ggf. Krankengymnastik nach Abklingen der akuten Beschwerden.
  - Nichtsteroidale Antirheumatika bei ausgeprägten Beschwerden.
  - Bei Stressfraktur stabilisierende Orthese für 12–24 Wochen mit absolutem Sportverbot, dann langsamer Belastungsaufbau mit physiotherapeutischer Begleitung zur Stabilisierung der Rumpfmuskulatur.
  - Bei asymptomatischer Olisthese Meyerding-Grad 1 und 2 ist Sport möglich, Risikosportarten vermeiden.

##### Operative Therapie.

- Bei Meyerding-Grad 1 und 2 nur bei persistierenden Beschwerden oder Progredienz der Olisthese.
- Direkte Osteosynthese des Pars-interarticularis-Defekts oder segmentale Fusion bei geringgradigem Gleitvorgang (bis Meyerding 1).
- Bei Kindern/Jugendlichen Pars-interarticularis-Repair versuchen:
  - Schraubenosteosynthese (Buck-Schraube)
  - Zuggurtungsosteosynthese (Edinburgh Repair nach Scott)
  - Hakenschraube nach Morscher
- Ab Meyerding 2 bei persistierenden Beschwerden, radikulären Beschwerden oder Progredienz des Abrutschs segmentale Fusion in-situ oder nach Reposition erwägen:
  - Fusion mit Pedikelschrauben und interkorporeller Fusion von ventral (ALIF), dorsal (TLIF) oder ventrodorsal möglich.
  - Eine Reposition vor Fusion geht mit einem ca. 30 % Risiko einer zumindest temporären Schädigung der Nervenwurzel L5 einher.

#### Welches Vorgehen empfehlen Sie im vorliegenden Fall und warum?

- Im vorliegenden Fall findet sich eine bereits weit fortgeschrittene Spondylolisthese Grad 4 nach Meyerding. Die Beschwerden nehmen im Verlauf eher zu, es besteht bereits eine radikuläre Beschwerdesymptomatik mit sensiblem Defizit. Zudem findet sich eine skoliotische Fehlhaltung, die aktuell nicht therapiebedürftig ist. Aufgrund der ausgeprägten Spondylolisthese mit beginnender neurologischer Symptomatik ist eine konservative Therapie in diesem Fall nicht indiziert, da hierunter mit einer Verschlechterung der Befunde zu rechnen ist. Daher wurde der Patientin und ihren Eltern die operative Therapie empfohlen.
- Ein operativer Pars-Repair ist aufgrund des vorangeschrittenen Befundes nicht indiziert. Ziel der operativen Therapie ist:
  - Das Aufhalten der progredienten Anterolisthese.
  - Die Reduktion der radikulären Symptomatik.
  - Die Rekonstruktion der sagittalen Balance und Besserung bzw. Aufhalten der Progredienz der skoliotischen Fehlhaltung.
- Zum Erreichen aller o. g. Ziele ist eine dorsale Instrumentation mit Pedikelschrauben und interkorporeller Fusion indiziert (Abb. 2a, b). Ein Repositionsversuch ist indiziert, um die ausgeprägte sagittale Dysbalance zu beheben. (Relordorsierung des Segmentes L5/S1)
- Neben den allgemeinen Operationsrisiken muss zwingend über das Risiko eines persistierenden Schadens der Nervenwurzel L5 aufgeklärt werden. Andere spezifische Risiken sind:
  - Scheitern der Reposition bzw. Repositionsverlust im Verlauf
  - persistierende lumbale und radikuläre Beschwerden
  - Pseudarthrose mit möglicher erneuter Operation
  - Progredienz der Skoliose trotz erfolgreicher Rekonstruktion der sagittalen Balance
- Postoperativ sollte eine stabilisierende Orthese für 12 Wochen verordnet werden. Physiotherapie sollte nach 10–12 Wochen begonnen werden. Kontaktsportarten sollten beschwerdeabhängig nach frühestens 6 Monate postoperativ wieder durchgeführt werden.
- Durchführung der radiologischen Kontrollen EOS-GWS erfolgt direkt postoperativ, sowie nach 3, 12 und 24 Monaten postoperativ, oder bei Beschwerden.

**Figure 2:**